# Readiness of Zanzibar’s public health facilities to deliver NCD services: a cross-sectional survey toward UHC

**DOI:** 10.1038/s41598-026-55541-6

**Published:** 2026-06-01

**Authors:** Omar Mwalim, Kjell Arne Johansson, Tarun Shankar Choudhary, Peter Hangoma

**Affiliations:** 1 Ministry of Health, Zanzibar, Tanzania; 2 Bergen Centre for Ethics and Priority Setting in Health, Department of Global Public Health and Primary Care, University of Bergen, Bergen, Norway; 3 Chr. Michelson Institute (CMI), Bergen, Norway; 4 University of Zambia, Lusaka, Zambia

**Keywords:** Noncommunicable diseases, Health services accessibility, Readiness assessment, Health facilities, Health care, Medical research

## Abstract

Non-communicable diseases (NCDs) are a growing public health challenge, particularly in low- and middle-income countries (LMICs), where weak health systems limit access to effective care. Achieving Universal Health Coverage (UHC) requires health facilities that are equipped and ready to deliver NCD services. However, evidence on the readiness of health facilities to provide these services in various contexts is limited. This study contributes to filling this gap by assessing the availability and readiness of health facilities to provide essential NCD services in the context of Zanzibar. We employed a facility cross-sectional survey and collected data across all 52 public and parastatal health facilities in Zanzibar using an adapted WHO Service Availability and Readiness Assessment (SARA) tool, incorporating elements from the Pen Plus survey and the Access Bottlenecks, Costs, and Equity (ABCE) survey tools. We generated readiness scores across various strata and used multiple regression analysis to examine factors associated with readiness. Readiness was assessed in terms of availability of trained workforce, essential medicines, diagnostic capacity, financial risk protection mechanisms, and patient registration and follow-up systems. We found an average readiness score of 54% across all facilities, with primary health care centers having the lowest readiness score (29%) compared to the referral hospital (73%). Readiness was particularly low in diagnostic and treatment services (40%), financing and payment mechanisms (31%), and essential medicines (53%), but comparatively higher in healthcare workforce (73%) and infrastructure readiness (69%). Lower-level facilities were associated with lower readiness scores after adjusting for key confounders in the multiple regression analysis. Availability of both inpatient and outpatient care was associated with significantly higher readiness (β = 10.50, *p* = 0.029). Primary health care facilities remain underprepared to deliver essential NCD services and overall readiness scores mask substantial variation by service type and facility. Strengthening service readiness will require targeted investments in workforce training, diagnostic capacity, supply chain systems, and financial protection measures to advance equitable NCD care as part of UHC efforts.

## Introduction

Non-communicable diseases (NCDs) such as hypertension, diabetes, cardiovascular diseases, cancers, and chronic respiratory illnesses are now the leading cause of death globally, accounting for approximately 70% of all deaths^1,2^ with over 75% of these occurring in low- and middle-income countries (LMICs)^3,4^. NCDs disproportionately affect working-age populations, contributing to lost productivity, high out-of-pocket expenditures, and broader economic instability. Projections estimate that the huge economic costs of NCDs will reverse health and development gains^5,6^. This poses a challenge for Sustainable Development Goal (SDG) 3^7^, particularly target 3.4, which aims to reduce premature mortality from NCDs through prevention and treatment. This calls for health systems that can deliver quality NCD care for all in need without imposes financial hardship in line with Universal Health Coverage (UHC)^8,9^.

However, health system readiness in many LMICs to deliver quality NCD care remains low, especially in primary care facilities which are the entry points for both prevention and management of NCDs^10^. Facilities in most LMICs often lack trained personnel, diagnostics, essential medicines, and structured follow-up systems in resource constrained settings^11,12^. A recent systematic review from sub-Saharan Africa, South Asia, and other LMICs found consistently limited availability of NCD-specific diagnostics, medicines, and infrastructure, alongside shortages of trained health workers and weak referral systems^13^, with variation in availability across service types and geographical areas. It is important to document which service types are most lacking in which contexts. In particular, there is limited evidence from smaller or decentralized health systems such as Zanzibar.

The most recent Zanzibar WHO STEPwise survey (2011) revealed a high prevalence of key risk factors: 33.3% of adults had hypertension, 24.5% had high cholesterol, 3.7% were diabetic, and 14.3% were obese. Nearly the entire adult population, 97.9%, fell short of recommended fruit and vegetable consumption^14^. In response, the Ministry of Health established a dedicated NCD Unit in 2012 and committed to decentralizing services from secondary and tertiary levels to primary care.

By 2021, the number of NCD clinics had expanded from 8 to 52, with nearly 80% operating at the primary level. This expansion signals strong policy commitment which has also been observed in other countries but also raises important questions about service quality and health system capacity. Are these facilities ready to deliver NCD care at scale? Are the essential building blocks like human resources, medicines, diagnostics, and referral systems in place to make services functional and equitable? Without such readiness, expanded service availability may have limited impact on outcomes.

To date, no study has assessed the readiness of Zanzibar’s public health facilities to deliver NCD services. This paper aims to fill this gap. Specifically, we will assess: (1) to what extent public and parastatal health facilities in Zanzibar are ready to deliver essential NCD services across key domains; (2) major readiness gaps, particularly at the primary care level; and (3) facility-level characteristics that are associated with higher or lower NCD readiness.

## Methods

### Study setting

The study was conducted in Zanzibar, a semi-autonomous country within the United Republic of Tanzania. Zanzibar comprises two main islands, Unguja and Pemba, with a population of approximately 1.8 million, growing at an annual rate of 3.1%^15^. The health system in Zanzibar is structured into three tiers: primary, secondary, and tertiary levels with a total of 364 health facilities, including 187 public, 156 private, and 21 parastatal facilities. However, not all of them provide NCD care. This study focused on public and parastatal facilities. The reason for not engaging private facilities in this study is that the health system has not yet fully integrated them, particularly in terms of data management, supervision, and overall operational governance. Among the 187 public and 21 parastatal facilities, only 52 offer NCD care.

### Study design and sampling

We employed a cross-sectional, facility-based design to assess the readiness of health facilities in Zanzibar to deliver NCD services. The study was a complete census of all 52 public and parastatal health facilities that provide at least one NCD-related service such as the diagnosis, treatment, or management of hypertension, diabetes, chronic respiratory disorders, or cardiovascular diseases at the time of data collection. Data were collected in both Unguja and Pemba Islands over a one-month period (March–April 2022) using a structured electronic questionnaire administered via tablets.

Because the study encompassed all eligible facilities, no sampling was undertaken. In a census design, all units in the target population are measured, eliminating sampling error and yielding the highest attainable precision for population-level estimates.

### Tool development, adaptation, and training

The survey instrument was adapted primarily from the WHO Service Availability and Readiness Assessment (SARA) tool^16^, retaining the core structure and tracer indicators. To enhance the comprehensiveness of the readiness assessment, we incorporated selected variables from the Access, Bottlenecks, Cost, and Equity (ABCE) survey^17^ and the PEN-Plus initiative^18^, focusing particularly on financing mechanisms and patient follow-up systems.

Readiness scoring followed the WHO SARA methodology. While the tool was not independently validated, the adaptation maintained the integrity of the original SARA methodology. Additions from ABCE were reviewed for consistency and piloted in three facilities before full deployment.

Data collectors received three days of intensive training covering study objectives, use of the digital data collection platform (Survey Solutions), operational definitions of tracer items, and ethical considerations. Supervisors conducted field monitoring and spot checks throughout the data collection period.

### Data collection and quality assurance

Data were collected electronically and uploaded in real time to a secure central server. Logic checks were embedded in the tool to prevent inconsistent or incomplete responses. Daily reviews were conducted by supervisors to ensure accuracy and completeness, and random verification visits were made to selected facilities to validate submitted responses. Data were uploaded to a secure Office of Chief Government Statistician server through the Survey Solution platform. No individual patient records or patient-level sociodemographic data were collected.

### Readiness scoring methodology

Readiness was assessed across eight domains: health workforce, diagnostic tools, essential medicines, equipment, infrastructure, patient education and support services, financial protection mechanisms, and registration/follow-up systems. For each domain, we calculated an overall mean readiness score for each facility level by averaging the proportion of facilities responding ‘Yes’ across all tracer items in that domain. When an item had multiple response categories (e.g., referral destinations to district, regional, or tertiary hospitals), each category was treated as a separate tracer item. Consequently, the number of tracer items varied by domain (range: 4–18). For each domain, the proportion of tracer items available was calculated as (n/N × 100), where n is the number of available tracer items and N represents the number of tracer items assessed within that domain. Tracer items were selected primarily from internationally standardized WHO SARA indicators and supplemented with selected PEN-Plus and ABCE items to capture broader NCD system capacity. Some advanced diagnostics and equipment indicators may exceed the routine scope of services expected at primary health care level in Zanzibar; therefore, readiness scores should be interpreted as measures of comparative system capacity rather than strict compliance with facility-level policy mandates.

Domain-specific scores were calculated as the proportion of tracer items available within each domain and the overall facility readiness scores was computed as the unweighted mean of the eight domain scores. This approach aligns with the WHO SARA methodology, while allowing for the inclusion of two additional domains using validated items from ABCE and PEN-Plus; however, this adaptation did not alter the scoring logic or apply differential weighting.

All tracer items within a domain were weighted equally across facility levels to allow standardized comparison of NCD service readiness across the health system. However, some tracer items particularly highly specialized human resources, diagnostics, or treatment services may be less applicable to lower-level primary care facilities and should therefore be interpreted as indicators of overall system capacity rather than strict compliance with level-specific service mandates.

### Statistical analysis

Descriptive statistics were used to summarize facility characteristics and readiness scores. To identify factors associated with readiness, we fitted a multiple linear regression model using facility readiness score as the dependent variable. Independent variables included facility type (referral, regional, district, primary, parastatal), catchment area (urban, peri-urban, rural), ownership (public or parastatal), operating hours, after-hours/weekend service availability, and type of care (inpatient vs. outpatient). The regression model was pre-specified, and multicollinearity was assessed using variance inflation factors (VIF), all of which were below the threshold of 5. Model diagnostics confirmed assumptions of linearity and homoscedasticity. We report beta coefficients with corresponding p-values and confidence intervals.

### Ethical approval

This study was conducted as part of a collaborative capacity-building project between the Ministry of Health, Zanzibar, and the University of Bergen. Ethical clearance was obtained from the Zanzibar Health Research Ethical Committee (ZAHREC), while the Regional Committees for Medical and Health Research Ethics in Western Norway (REK-VEST) granted an exemption, as the study involved no direct human interaction.

## Results

### Facility characteristics

NCD service delivery in Zanzibar is dominated by primary health care centers, with relatively broad distribution across rural, peri-urban and urban settings. Operational arrangements varied across facilities, including differences in working hours and after-hours service provision. Detailed facility characteristics are presented in Table 1.

**Table 1:** Facility characteristic.

Facility type	*N* (%)
Referral hospital	1 (2)
Regional hospital	1 (2)
District hospital	5 (10)
Primary health care center	41 (79)
Parastatal	4 (8)
Catchment area type of the facility	
Urban	15 (29)
Peri-Urban	17 (33)
Rural	20 (38)
Average working hours per day	
8 h	27 (52)
12 h	5 (10)
24 h	20 (38)
Availability of services	
After hours only	18 (35)
After hours and weekend care	24 (46)
Neither after working hours nor weekend	10 (19)

### General readiness

Overall readiness for NCD service delivery was moderate, with substantial variation across domains and facility types (Table 2). Healthcare workforce and infrastructure showed comparatively higher readiness, whereas diagnostic and treatment services, NCD education and support services, and financing mechanisms remained limited. Primary health care centers consistently demonstrated the lowest readiness across most domains, highlighting major frontline service delivery gaps.

**Table 2:** Mean readiness scores of NCD service delivery by facility type across key domains.

Domain	Referral hospital (%) [*n* = 1]	Regional hospital (%) [*n* = 1]	District hospital (%) [*n* = 5]	Primary health care center (%) [*n* = 41]	Parastatal (%) [*n* = 4]	Mean readiness (%)
Healthcare workforce	94	82	76	39	74	73
Essential medicines	78	43	52	27	64	53
Medical equipment	80	72	53	24	63	58
Diagnostic & treatment services	38	44	48	20	48	40
NCD education & support services	75	75	30	23	25	46
Registration, follow-up & referral	67	56	40	47	56	53
Facility infrastructure	89	65	64	45	81	69
Financing and payment mechanism	67	8	13	0	66	31
Overall mean readiness (%)	73	57	48	29	61	54

Readiness also differed substantially by level of care. Referral and regional hospitals demonstrated higher overall readiness, while primary health care centers consistently showed the lowest scores across most domains. Given that primary health care centers constitute the majority of NCD service delivery points, these findings highlight important frontline service delivery gaps within the health system.

### Condition-specific readiness for hypertension, diabetes, and respiratory disease

Drug, diagnostic and equipment readiness for management of hypertension, diabetes and respiratory diseases varied across levels of care, with referral hospitals consistently having the highest readiness for hypertension, diabetes, and respiratory care, while primary health care centers showed major gaps across all domains. (Table 3).

**Table 3:** Facility readiness for managing hypertension, diabetes, and respiratory disease by domain.

Condition	Drug readiness (%)	Diagnostic readiness (%)	Equipment readiness (%)
Hypertension			
Primary health care center	32	33	35
District hospital	60	56	60
Regional hospital	79	71	80
Referral hospital	85	88	91
Parastatal facility	64	61	55
Diabetes			
Primary health care center	5	25	27
District hospital	100	60	60
Regional hospital	90	77	83
Referral hospital	100	94	97
Parastatal facility	52	75	64
Respiratory disease			
Primary health care center	10	20	56
District hospital	0	15	60
Regional hospital	55	48	73
Referral hospital	71	83	89
Parastatal facility	100	56	100

The largest readiness gaps were observed for diabetes and respiratory disease services.

### Determinants of health facility readiness for NCD services

Multiple regression analysis identified facility type as the strongest predictor of readiness for NCD service delivery (Table 4). Compared with referral hospitals, primary health care centers and district hospitals had significantly lower readiness scores. Geographic location and ownership status were not significantly associated with readiness, while facilities offering both inpatient and outpatient services demonstrated higher readiness. Overall, the model explained a substantial proportion of the variation in readiness scores.

**Table 4:** Multivariate regression of predictors of facility readiness for NCD services.

	Estimate	Std. error	t-value	*p*-value	Significance
(Intercept)	59.4984	7.1774	8.290	3.90e-10	***
Facility type					
Regional hospital	−14.1708	8.6494	−1.638	0.10939	
District hospital	−20.5785	6.7727	−3.038	0.00423	**
Primary health care center	−32.3524	6.8711	−4.708	3.13e-05	***
Parastatal facility	−15.3969	7.4906	−2.056	0.04657	*
Catchment area					
Peri-Urban	1.2521	2.6975	0.464	0.64511	
Rural	−1.5905	2.7734	−0.573	0.56961	
Ownership					
Parastatal ownership	11.0936	6.6845	1.660	0.10502	
Operating hours					
12 h	0.8124	3.4224	0.237	0.81361	
24 h	−4.4584	4.4581	−1.000	0.32351	
After hours availability					
After hours and weekend care	3.0832	3.6060	0.855	0.39776	
Neither	5.2124	2.4196	2.154	0.03745	*
Type of care offered					
Both in and outpatient care	10.4989	4.6249	2.270	0.02881	*

Significance levels: ***p < 0.001; **p < 0.01; *p < 0.05.

Geographical location, ownership status and operating hours were not significantly associated with readiness, whereas facilities offering both inpatient and outpatient services demonstrated higher readiness.

## Discussion

Primary health care centers in Zanzibar were substantially less prepared to deliver essential NCD services than higher-level facilities, despite serving as the main entry point for most patients. Although Zanzibar has expanded NCD clinics from 8 to 52 facilities over recent years, these services remain available in only about one-quarter of all public and parastatal health facilities in the health system. This limited service coverage represents an important access gap, particularly in the context of the growing burden of hypertension, diabetes, obesity, and cardiovascular disease in Zanzibar^19^. The findings suggest that expansion of NCD services has not yet been matched by sufficiently broad integration of NCD care within frontline primary care facilities. Expanding the geographical availability and functional capacity of NCD services therefore remains a critical priority for achieving equitable UHC. Expanding the geographical availability of functional NCD services remains a critical priority for achieving equitable UHC. Similar readiness gaps at the frontline level have been documented across LMICs, underscoring the systemic challenges of building functional NCD care capacity where it is most needed.

Similar findings have also been reported in Kenya, where a national survey demonstrated substantial gaps in readiness for NCD service delivery at primary care level, particularly in diagnostics and medicine availability^20^. Rogers et al. further found in Uganda that most lower-level public facilities lacked essential WHO-PEN diagnostic and treatment capacity for NCD care, reinforcing the broader regional pattern of underprepared frontline services^21^. Evidence from Sidama, Ethiopia, only 29% of PHC units were ready to manage hypertension and 28% to manage diabetes, with readiness strongly influenced by the availability of trained staff, particularly in rural areas^22^. Likewise, the National Noncommunicable Disease Monitoring Survey (NNMS) in India reported critical shortages of nurse-midwives and health assistants at PHC level, directly undermining service readiness^23^. The global systematic review by Kabir et al. (2022) confirmed that workforce availability is a persistent bottleneck for NCD care, especially for chronic conditions requiring sustained engagement and continuity of care^13^. These patterns suggest that expanding service availability without simultaneously addressing staffing gaps particularly at the primary level will deliver limited improvements in readiness and outcomes.

The workforce gap is compounded by inadequate diagnostic capacity. In Zanzibar, PHCs scored only 40% in this domain, reflecting a pattern seen elsewhere. In India, fewer than 3% of PHCs had ECGs or serum creatinine analyzers, and even basic tools like glucometers were often absent^24^. Ethiopian facilities without essential diagnostics were far less likely to be rated “ready” for NCD care, with their absence linked to more referrals and higher patient costs^22^. Kabir et al. (2022) identified diagnostic capacity as the weakest domain in nearly all reviewed studies, citing underfunding, equipment breakdowns, and weak logistics systems as key causes^13^. In Bangladesh, diagnostic facilities for cervical cancer were concentrated almost entirely in tertiary centres, leaving lower-level facilities unable to perform even basic screening^25^. Without reliable diagnostic capability, early detection is compromised, referrals become the default, and health systems cannot achieve UHC targets for chronic disease management.

Essential medicines and equipment also remain a major constraint. In Zanzibar, tracer NCD medicines were inconsistently available, particularly in PHCs. This mirrors findings from India, where full availability of essential NCD medicines was found in just 1.1% of rural public PHCs, with notable gaps in antihypertensives and insulin^23^. In Ethiopia, fewer than one-third of facilities stocked the expected range of antihypertensive or antidiabetic drugs, and medicine availability was closely correlated with readiness scores^22^. Kabir et al. (2022) observed that essential medicines and functional equipment consistently scored lowest across countries, with stock-outs, fragmented procurement, and inadequate maintenance among the main drivers^13^. In Bangladesh, only tertiary and specialised hospitals surpassed the 70% readiness threshold for cervical cancer services, largely due to stronger supply chain systems and equipment upkeep^25^. These patterns point to the need for centralised procurement, robust buffer stocks, and active monitoring of supply and functionality, especially in rural and hard-to-reach areas.

Although financing arrangements were not the main focus of this study, modest readiness differences between parastatal and fully public facilities in Zanzibar suggest that governance and resource management structures matter. Kabir et al. (2022) noted that fragmented, underfunded primary care systems which are often under direct public management, tend to be slower to adopt innovations or maintain steady NCD supplies^26^. In India, programmatic funding under NPCDCS had not consistently translated into readiness due to implementation bottlenecks and weak budget execution^23^, and in Ethiopia, similar patterns were observed^22^. In Bangladesh, despite national policies for cancer services, financial and infrastructural readiness was concentrated in select tertiary centres^25^. These experiences highlight the importance of improving fund flow mechanisms, accountability, and facility-level autonomy for resource use.

Regression analysis in Zanzibar reinforced these patterns, showing that PHC consistently was negatively associated with readiness, even after adjusting for catchment size and ownership. Facilities offering both inpatient and outpatient services scored significantly higher, consistent with findings from Sidama, Ethiopia, where primary hospitals outperformed health centres due to broader service mandates and greater infrastructure^22^. Interestingly, facilities without extended hours scored slightly higher than those open 24/7, echoing Kabir et al.’s (2022) observation that overstretched facilities may experience resource dilution and staff burnout, ultimately lowering quality^13^. This seemingly counterintuitive finding may reflect operational trade-offs: facilities with limited hours, often PHCs, may better maintain essential supplies and staff focus within constrained service windows, whereas 24-hour facilities frequently manage higher patient volumes without proportional increases in capacity, leading to diluted readiness across domains. These results suggest that expanding service hours without matching investments in staffing, equipment, and systems risks undermining overall preparedness.

Taken together, the multi-country evidence points to three urgent priorities for Zanzibar. First, investments in primary care must target functionality, not just coverage but also strengthening the workforce, building diagnostic platforms, and ensuring reliable medicine supply. Second, structured facility-level performance monitoring, as recommended by Krishnan et al. (2021)^23^, coupled with greater managerial autonomy, can help close operational gaps. Third, decentralisation must be underpinned by sustainable financing, robust supply chains, and effective referral systems that match the realities of frontline care. The convergence of evidence from Ethiopia^22^, India^23^, Bangladesh^25^, and the global literature^13^ underlines that these challenges are not unique to Zanzibar. Addressing them is essential to strengthen readiness at the primary level not only to expand access, but also to secure quality, continuity, and resilience in NCD care.

### Limitations and equity considerations

Several limitations warrant caution. First, data on tracer items were self-reported by facility staff, raising the risk of social desirability bias and potential overreporting of service availability. Second, we did not triangulate readiness data with patient-level outcomes, quality measures, or service utilization, which limits our ability to assess effective coverage. Third, the study did not include private-sector facilities, which may provide a substantial share of chronic disease care and thus limits generalizability across the entire health system. Lastly, while the tool adapted WHO SARA and PEN-Plus methods, some domain-specific indicators may not fully capture quality or reliability of services (e.g., presence vs. functionality of diagnostic equipment). The use of standardized tracer items across all facility levels may underestimate readiness among lower-level facilities, particularly for domains that include highly specialized diagnostics, equipment, human resources, or treatment services not routinely expected at primary health care level within the Zanzibar health system. Consequently, lower readiness scores in such facilities should be interpreted cautiously, as they may reflect the inclusion of higher-level service capacity indicators within a standardized assessment framework rather than strict non-compliance with level-specific service expectations. Although data collection was conducted in 2022, the findings remain relevant given the limited availability of comprehensive system-wide assessments of NCD service readiness in Zanzibar and the persistence of many identified structural gaps within ongoing health system reforms. Despite these limitations, the study offers robust, system-wide evidence that can guide targeted investments and policy reforms to strengthen NCD readiness in Zanzibar.

## Conclusion

This study highlights critical gaps in the readiness of Zanzibar’s health facilities to provide NCD services, especially at the primary care level where most patients seek care. Although workforce and infrastructure were relatively strong, essential medicines, diagnostics, and follow-up systems were severely limited, reflecting the gap between nominal availability and functional service delivery. By contrast, higher-level and parastatal facilities were better equipped, and facilities offering both inpatient and outpatient care demonstrated stronger readiness overall. Addressing these disparities will require targeted investments in PHCs, improved supply chains, and strengthened referral and financing mechanisms. The findings provide actionable evidence to guide policy and planning toward more equitable and effective NCD care in Zanzibar.

## Data Availability

The datasets used and/or analysed during the current study are available from the corresponding author on reasonable request.

## Annex 1: detailed breakdown of scores across all the eight domains

### Interpretation note

The detailed domain-level tables in the annex should be interpreted with caution. Some apparent mismatches across domains (e.g., equipment present but services not offered, or community health workers available but few education programs reported) reflect systemic implementation gaps rather than data inconsistencies. In several cases, tracer items were reported as “available” in terms of physical presence, but functionality, consumables, or trained personnel were lacking, preventing their routine use. Similarly, reported insurance participation at referral or parastatal facilities does not always translate into patient-level financial protection, as arrangements often operate through supply-side reimbursements or mixed financing models. These nuances highlight a critical distinction between nominal availability and effective readiness, reinforcing the study’s central finding that service functionality at primary health care level remains severely constrained despite expansions in coverage.

**Domain 1:** Human resource. Overall mean readiness for each facility level was calculated by averaging the percentage of facilities responding ‘Yes’ across all tracer items within this domain (n = 17 items).

Cadre	Referral hospital, *N* = 1	Regional hospital, *N* = 1	District hospital, *N* = 5	Primary health care center, N = 41	Parastatal, *N* = 4	Mean readiness
General practitioners (GP)	1 (100%)	1 (100%)	5 (100%)	4 (9.8%)	3 (75%)	77%
Resident doctors	1 (100%)	0 (0%)	1 (20%)	0 (0%)	2 (50%)	34%
Cardiologists	1 (100%)	1 (100%)	1 (20%)	0 (0%)	1 (25%)	49%
Cardiac surgeons	1 (100%)	0 (0%)	0 (0%)	0 (0%)	0 (0%)	20%
Other specialists	1 (100%)	1 (100%)	3 (60%)	2 (4.9%)	2 (50%)	63%
Health officers	1 (100%)	1 (100%)	5 (100%)	27 (66%)	4 (100%)	93%
Nurses (Psyc, Gen, Asst, Officer)	1 (100%)	1 (100%)	5 (100%)	34 (83%)	4 (100%)	97%
Midwives	1 (100%)	1 (100%)	4 (80%)	27 (66%)	2 (50%)	79%
Pharmacist	1 (100%)	1 (100%)	5 (100%)	4 (9.8%)	3 (75%)	77%
Registration staff	1 (100%)	1 (100%)	4 (80%)	3 (7.3%)	3 (75%)	72%
Laboratory technician	1 (100%)	1 (100%)	5 (100%)	33 (80%)	4 (100%)	96%
Community health workers	0 (0%)	0 (0%)	2 (40%)	22 (54%)	2 (50%)	29%
Pharmaceutical technician	1 (100%)	1 (100%)	5 (100%)	21 (51%)	4 (100%)	90%
Assistance medical officers	1 (100%)	1 (100%)	5 (100%)	3 (7.3%)	4 (100%)	81%
Clinical officer	1 (100%)	1 (100%)	5 (100%)	36 (88%)	4 (100%)	98%
Dental technician	1 (100%)	1 (100%)	5 (100%)	19 (46%)	4 (100%)	89%
Others	1 (100%)	1 (100%)	5 (100%)	40 (98%)	4 (100%)	100%
Overall mean readiness	94%	82%	76%	39%	74%	73%

**Domain 2:** Essential medicines. Overall mean readiness for each facility level was calculated by averaging the percentage of facilities responding ‘Yes’ across all tracer items within this domain (n = 37 items).

Medicine	Referral hospital, *N* = 1	Regional hospital, *N* = 1	District hospital, *N* = 5	Primary health care center, *N* = 41	Parastatal, *N* = 4	Mean readiness
Penicillin (Amoxicillin, Pen V, X-pen)	1 (100%)	1 (100%)	4 (80%)	30 (73%)	3 (75%)	86%
Short-acting insulin (soluble insulin)	0 (0%)	1 (100%)	5 (100%)	2 (4.9%)	1 (25%)	46%
Intermediate-acting insulin (Novolin N)	1 (100%)	0 (0%)	2 (40%)	0 (0%)	0 (0%)	28%
Long-acting insulin (Lente insulin)	0 (0%)	1 (100%)	4 (80%)	2 (4.9%)	2 (50%)	47%
Metformin	1 (100%)	1 (100%)	3 (60%)	24 (59%)	4 (100%)	84%
Sulfonylureas (Glyburide, Glimepiride)	1 (100%)	0 (0%)	2 (40%)	10 (24%)	2 (50%)	43%
Aspirin	1 (100%)	1 (100%)	5 (100%)	22 (54%)	4 (100%)	91%
Loop diuretics (Furosemide-Lasix)	1 (100%)	1 (100%)	5 (100%)	36 (88%)	4 (100%)	98%
Angiotensin-converting enzyme (ACE) inhibitors (Captopril)	1 (100%)	0 (0%)	3 (60%)	13 (32%)	4 (100%)	58%
Beta-blockers (Atenolol, Propranolol)	1 (100%)	1 (100%)	3 (60%)	18 (44%)	3 (75%)	76%
Potassium-sparing diuretic (spironolactone-Aldactone)	1 (100%)	0 (0%)	1 (20%)	0 (0%)	2 (50%)	34%
Thiazide diuretics (Aprinox)	1 (100%)	0 (0%)	3 (60%)	21 (51%)	4 (100%)	62%
Calcium channel blockers (Amlodipine)	1 (100%)	0 (0%)	3 (60%)	13 (32%)	4 (100%)	58%
Methyldopa (Aldomet)	1 (100%)	0 (0%)	3 (60%)	13 (32%)	4 (100%)	58%
Nitrates (Isosorbide Dinitrate)	1 (100%)	0 (0%)	1 (20%)	0 (0%)	2 (50%)	34%
Heparin	0 (0%)	0 (0%)	1 (20%)	0 (0%)	0 (0%)	4%
Warfarin	0 (0%)	1 (100%)	2 (40%)	0 (0%)	0 (0%)	28%
Other (non-warfarin) oral anti-coagulant (e.g. enoxaparin, rivaroxaban)	0 (0%)	0 (0%)	1 (20%)	0 (0%)	0 (0%)	4%
Potassium, oral	0 (0%)	0 (0%)	1 (20%)	1 (2.4%)	0 (0%)	4%
Proton-pump inhibitors (Omeprazole, Lansoprazole)	1 (100%)	0 (0%)	1 (20%)	19 (46%)	4 (100%)	53%
Lactulose	0 (0%)	0 (0%)	1 (20%)	0 (0%)	2 (50%)	14%
Statins -Atorvastatin (Lipitor)	0 (0%)	1 (100%)	3 (60%)	0 (0%)	2 (50%)	42%
Hydralazine	1 (100%)	1 (100%)	4 (80%)	6 (15%)	2 (50%)	69%
Isosorbide dinitrate	1 (100%)	0 (0%)	1 (20%)	0 (0%)	2 (50%)	34%
Hydroxyurea (Hydroxycarbamide)	1 (100%)	0 (0%)	1 (20%)	0 (0%)	0 (0%)	24%
Prophylactic antibiotics (Doxycycline)	1 (100%)	0 (0%)	4 (80%)	24 (59%)	4 (100%)	68%
Inhaled corticosteroids (Beclomethasone)	1 (100%)	0 (0%)	1 (20%)	2 (4.9%)	3 (75%)	40%
Aminophylline	1 (100%)	1 (100%)	2 (40%)	8 (20%)	3 (75%)	67%
Oral corticosteroids (Prednisolone, Dexamethasone)	1 (100%)	1 (100%)	3 (60%)	13 (32%)	4 (100%)	78%
Oral morphine or other opioids (Tramadol, Codeine)	1 (100%)	0 (0%)	2 (40%)	3 (7.3%)	3 (75%)	44%
Anti-emetics (Metoclopramide, Promethazine)	1 (100%)	1 (100%)	2 (40%)	17 (41%)	4 (100%)	76%
Anti-depressants (Amitriptyline)	1 (100%)	0 (0%)	3 (60%)	13 (32%)	2 (50%)	48%
Anti-psychotics- Chlorpromazine (CPZ), Olanzapine	1 (100%)	1 (100%)	3 (60%)	13 (32%)	2 (50%)	68%
Laxatives – Bisacodyl (Dulcolax)	1 (100%)	0 (0%)	1 (20%)	0 (0%)	3 (75%)	39%
Analgesics (Paracetamol, Diclofenac)	1 (100%)	1 (100%)	5 (100%)	39 (95%)	4 (100%)	99%
Topical antifungal (Miconazole, Clotrimazole)	1 (100%)	0 (0%)	2 (40%)	17 (41%)	4 (100%)	56%
Cotrimoxazole	1 (100%)	1 (100%)	5 (100%)	36 (88%)	4 (100%)	98%
Overall mean readiness	78%	43%	52%	27%	64%	53%

**Domain 3:** Medical equipment. Overall mean readiness for each facility level was calculated by averaging the percentage of facilities responding ‘Yes’ across all tracer items within this domain (n = 30 items).

Equipment	Referral hospital, *N* = 1	Regional hospital, *N* = 1	District hospital, *N* = 5	Primary health care center, *N* = 41	Parastatal, *N* = 4	Mean rediness
Radiography (X-ray)	1 (100%)	1 (100%)	5 (100%)	1 (2.5%)	2 (50%)	71%
Blood pressure measuring devices	1 (100%)	1 (100%)	5 (100%)	41 (100%)	4 (100%)	100%
Weight scale	1 (100%)	1 (100%)	5 (100%)	41 (100%)	4 (100%)	100%
Measuring tape or ruler	1 (100%)	1 (100%)	5 (100%)	41 (100%)	4 (100%)	100%
Hemoglobin	1 (100%)	1 (100%)	5 (100%)	35 (85%)	4 (100%)	97%
Erythrocyte sedimentation rate	1 (100%)	1 (100%)	5 (100%)	1 (2.4%)	3 (75%)	75%
Serum electrolytes	1 (100%)	1 (100%)	1 (20%)	0 (0%)	1 (25%)	49%
Creatinine	1 (100%)	1 (100%)	2 (40%)	0 (0%)	2 (50%)	58%
Coagulation (prothrombin time (PT)/INR)	1 (100%)	0 (0%)	1 (20%)	0 (0%)	2 (50%)	34%
Blood glucose	1 (100%)	1 (100%)	3 (60%)	20 (49%)	3 (75%)	77%
Monofilament	1 (100%)	0 (0%)	0 (0%)	20 (51%)	3 (75%)	45%
Hemoglobin A1c	1 (100%)	1 (100%)	1 (20%)	1 (2.5%)	2 (50%)	55%
C-peptide	0 (0%)	0 (0%)	0 (0%)	0 (0%)	0 (0%)	0%
Urine ketone testing	1 (100%)	1 (100%)	5 (100%)	10 (24%)	4 (100%)	85%
Electrocardiography	1 (100%)	1 (100%)	1 (33%)	0 (0%)	1 (25%)	52%
Ultrasound equipment with cardiac probes	1 (100%)	1 (100%)	1 (33%)	0 (0%)	1 (25%)	52%
Ultrasound equipment with abdominal probes	1 (100%)	1 (100%)	3 (100%)	7 (17%)	4 (100%)	83%
Liver function tests	1 (100%)	1 (100%)	3 (60%)	0 (0%)	3 (75%)	67%
Hepatitis B and C testing	1 (100%)	1 (100%)	5 (100%)	5 (12%)	4 (100%)	82%
Blood smear	1 (100%)	1 (100%)	5 (100%)	16 (39%)	3 (75%)	83%
Full blood count	1 (100%)	1 (100%)	5 (100%)	1 (2.4%)	3 (75%)	75%
Peak flow meters	0 (0%)	0 (0%)	0 (0%)	5 (12%)	1 (25%)	7%
Inhalers	0 (0%)	0 (0%)	0 (0%)	4 (9.8%)	4 (100%)	22%
Spacers	0 (0%)	0 (0%)	0 (0%)	0 (0%)	2 (50%)	10%
Spirometry	0 (0%)	0 (0%)	0 (0%)	0 (0%)	0 (0%)	0%
Nebulizers	1 (100%)	1 (100%)	3 (60%)	23 (56%)	4 (100%)	83%
Nasogastric tube	1 (100%)	1 (100%)	3 (75%)	6 (15%)	3 (75%)	73%
Bladder catheter	1 (100%)	1 (100%)	2 (50%)	20 (49%)	3 (75%)	75%
Opioid lock box	1 (100%)	0 (NA%)	1 (25%)	0 (0%)	0 (0%)	31%
Pressure-reducing mat	0 (0%)	0 (0%)	0 (0%)	0 (0%)	1 (25%)	5%
Overall mean readiness	80%	72%	53%	24%	63%	58%

**Domain 4:** Diagnostic and treatment services. Overall mean readiness for each facility level was calculated by averaging the percentage of facilities responding ‘Yes’ across all tracer items within this domain (n = 53 items).

Diagnostic/treatment	Referral hospital, *N* = 1	Regional hospital, *N* = 1	District hospital, *N* = 5	Primary health care center, *N* = 41	Parastatal, *N* = 4	Mean readiness
Abdominal ultrasound	0 (0%)	1 (100%)	5 (100%)	1 (2.4%)	3 (75%)	55%
Angiotensin-converting enzyme (ACE) inhibitor management	1 (100%)	0 (NA%)	4 (80%)	14 (34%)	4 (100%)	79%
Antibiotic therapy	1 (100%)	0 (NA%)	5 (100%)	35 (85%)	4 (100%)	96%
Anti-seizure medication management	0 (0%)	0 (NA%)	2 (50%)	10 (24%)	3 (75%)	37%
Any diagnostic testing for sickle cell	0 (0%)	0 (NA%)	2 (40%)	0 (0%)	1 (25%)	16%
Aspirin management	1 (100%)	0 (NA%)	5 (100%)	23 (58%)	4 (100%)	90%
Beta blocker management	1 (100%)	0 (NA%)	5 (100%)	18 (44%)	4 (100%)	86%
BiPAP	0 (0%)	0 (NA%)	0 (0%)	0 (0%)	1 (25%)	6%
Blood glucose testing	1 (100%)	1 (100%)	3 (60%)	30 (75%)	4 (100%)	87%
Blood transfusion	0 (0%)	1 (100%)	3 (60%)	1 (2.4%)	3 (75%)	47%
Bronchodilator and steroid inhaler management	0 (0%)	0 (NA%)	2 (40%)	8 (20%)	4 (100%)	40%
Chest X-ray	0 (0%)	1 (100%)	5 (100%)	2 (4.9%)	2 (50%)	51%
Chronic oral therapy (e.g., tamoxifen)	0 (0%)	0 (NA%)	0 (0%)	0 (0%)	0 (0%)	0%
CKD screening	0 (0%)	1 (100%)	3 (60%)	1 (2.4%)	1 (25%)	37%
C-peptide testing	0 (0%)	0 (NA%)	0 (0%)	0 (0%)	0 (0%)	0%
Diuretic management (e.g., furosemide, spironolactone)	1 (100%)	1 (100%)	5 (100%)	33 (83%)	4 (100%)	97%
Diuretic management	1 (100%)	0 (0%)	2 (40%)	9 (24%)	2 (50%)	43%
ECG	1 (100%)	1 (100%)	2 (40%)	2 (5.0%)	1 (25%)	54%
Hemoglobin A1c testing	1 (100%)	1 (100%)	0 (0%)	0 (0%)	1 (25%)	45%
Hepatitis C treatment	0 (0%)	0 (0%)	0 (0%)	1 (2.4%)	2 (50%)	10%
Hypertension screening	1 (100%)	1 (100%)	5 (100%)	40 (98%)	4 (100%)	100%
Imaging for diagnosis (CT) or MRI	0 (0%)	0 (0%)	0 (0%)	0 (0%)	0 (0%)	0%
Initiation and monitoring of hydroxyurea	0 (0%)	0 (NA%)	0 (0%)	0 (0%)	0 (0%)	0%
Insulin management	1 (100%)	1 (100%)	5 (100%)	7 (17%)	1 (25%)	68%
International normalized ratio (INR) testing	0 (0%)	0 (NA%)	1 (20%)	0 (0%)	0 (0%)	5%
Lactulose management	0 (0%)	0 (NA%)	0 (0%)	0 (0%)	1 (25%)	6%
Liver enzyme testing	0 (0%)	0 (0%)	3 (60%)	0 (0%)	2 (50%)	22%
Medical management with anti-hypertensives in 3 or more classes	1 (100%)	1 (100%)	5 (100%)	21 (51%)	3 (75%)	85%
Medication management for cirrhosis	0 (0%)	0 (0%)	0 (0%)	0 (0%)	1 (25%)	5%
Metformin management	1 (100%)	1 (100%)	5 (100%)	29 (71%)	4 (100%)	94%
Monofilament testing	1 (100%)	1 (100%)	2 (40%)	11 (28%)	2 (50%)	64%
Oral steroid management	1 (100%)	0 (0%)	4 (80%)	9 (22%)	2 (50%)	50%
Other symptom management	1 (100%)	0 (0%)	2 (40%)	10 (24%)	3 (75%)	48%
Oxygen	0 (0%)	0 (0%)	1 (20%)	5 (12%)	2 (50%)	16%
Pain management, non-opioids	1 (100%)	1 (100%)	5 (100%)	31 (78%)	4 (100%)	96%
Pain management, opioids	0 (0%)	0 (0%)	2 (40%)	6 (15%)	2 (50%)	21%
Physical therapy	0 (0%)	0 (0%)	3 (60%)	3 (7.3%)	1 (25%)	18%
Platelet testing	0 (0%)	0 (0%)	1 (20%)	0 (0%)	1 (25%)	9%
Propranolol management	0 (0%)	0 (NA%)	1 (20%)	2 (5.0%)	2 (50%)	19%
Respirator	0 (0%)	0 (NA%)	0 (0%)	0 (0%)	0 (0%)	0%
Retinal screening	0 (0%)	0 (0%)	4 (80%)	9 (22%)	2 (50%)	30%
Serum creatinine testing,	0 (0%)	0 (0%)	4 (80%)	0 (0%)	1 (25%)	21%
Spirometry	0 (0%)	0 (NA%)	0 (0%)	0 (0%)	0 (0%)	0%
Steroid inhaler management	0 (0%)	0 (0%)	2 (40%)	6 (15%)	3 (75%)	26%
Sulfonyl urea management	1 (100%)	0 (0%)	2 (40%)	4 (9.8%)	2 (50%)	40%
Surgical referral	1 (100%)	1 (100%)	4 (80%)	11 (27%)	4 (100%)	81%
Transcranial Doppler ultrasound,	1 (100%)	0 (0%)	0 (0%)	0 (0%)	0 (0%)	20%
Ultrasonography with abdominal probes	0 (0%)	0 (0%)	2 (40%)	0 (0%)	1 (25%)	13%
Ultrasonography with cardiac probes	0 (0%)	0 (0%)	1 (20%)	0 (0%)	1 (25%)	9%
Urine glucose testing	0 (0%)	1 (100%)	3 (60%)	11 (27%)	2 (50%)	47%
Warfarin or other anticoagulation management	0 (0%)	0 (NA%)	0 (0%)	0 (0%)	1 (25%)	6%
Mental health management	0 (0%)	0 (0%)	4 (80%)	15 (37%)	1 (25%)	28%
Neurological disorders (Epilepsy, Migraine, Dementia	0 (0%)	0 (0%)	4 (80%)	16 (39%)	1 (25%)	29%
Overall mean readiness	38%	44%	48%	20%	48%	40%

**Domain 5:** NCD education and support services. Overall mean readiness for each facility level was calculated by averaging the percentage of facilities responding ‘Yes’ across all items within the domain (n = 4 items).

Area	Referral hospital, *N* = 1	Regional hospital, *N* = 1	District hospital, *N* = 5	Primary health care center, *N* = 41	Parastatal, *N* = 4	Mean readiness
Are NCD-related educational materials available to patients at this facility?						
Yes	1 (100%)	1 (100%)	5 (100%)	19 (46%)	1 (25%)	74%
No	0 (0%)	0 (0%)	0 (0%)	22 (54%)	3 (75%)	26%
Is there NCD-related community education program organized by this facility?						
Yes	1 (100%)	1 (100%)	1 (20%)	13 (32%)	0 (0%)	50%
No	0 (0%)	0 (0%)	4 (80%)	28 (68%)	4 (100%)	50%
Is social support available to NCD patients at this facility?						
Yes	0 (0%)	0 (0%)	0 (0%)	6 (15%)	3 (75%)	18%
No	1 (100%)	1 (100%)	5 (100%)	35 (85%)	1 (25%)	82%
Are patients with type 1 diabetes provided with supplies for home management (e.g., home glucometer, testing strips, syringes, ketone strips)?						
Yes	1 (100%)	1 (100%)	0 (0%)	0 (0%)	0 (0%)	40%
No	0 (0%)	0 (0%)	5 (100%)	41 (100%)	4 (100%)	60%
Overall mean readiness	75%	75%	30%	23%	25%	46%

**Domain 6:** Registration, follow up and referral. Overall mean readiness for each facility level was calculated by averaging the percentage of facilities responding ‘Yes’ across all items within this domain (n = 12 items, with referral destinations i.e. district, regional, and tertiary were treated as separate indicators).

Area	Referral hospital, *N* = 1	Regional hospital, *N* = 1	District hospital, *N* = 5	Primary health care center, *N* = 41	Parastatal, *N* = 4	Mean readiness
Are patients formally enrolled or registered into care in this clinic?						
Yes	1 (100%)	1 (100%)	5 (100%)	41 (100%)	4 (100%)	100%
Do NCD patients attend routine follow-up visits at this clinic?						
Yes	1 (100%)	1 (100%)	4 (80%)	39 (95%)	4 (100%)	95%
No	0 (0%)	0 (0%)	1 (20%)	2 (4.9%)	0 (0%)	5%
Do patients receive follow up appointment dates?						
Yes	1 (100%)	1 (100%)	5 (100%)	41 (100%)	4 (100%)	100%
Does this facility have an established definition or criteria for NCD patients considered lost to follow-up?						
Yes	0 (0%)	0 (0%)	1 (20%)	8 (20%)	1 (25%)	13%
No	1 (100%)	1 (100%)	4 (80%)	33 (80%)	3 (75%)	87%
Is there a system in place at this facility to identify patients who have missed follow-up visits or are considered lost to follow-up?						
Yes	1 (100%)	0 (0%)	1 (20%)	8 (20%)	2 (50%)	38%
No	0 (0%)	1 (100%)	4 (80%)	33 (80%)	2 (50%)	82%
Is there a system in place at this facility to contact patients who have missed follow-up visits or are considered lost to follow-up?						
Yes	1 (100%)	1 (100%)	0 (0%)	8 (20%)	1 (25%)	49%
No	0 (0%)	0 (0%)	5 (100%)	33 (80%)	3 (75%)	51%
In case of referral, where do patients from this facility referred to for higher level care?						
District hospital	0 (0%)	0 (0%)	0 (0%)	19 (46%)	0 (0%)	9%
Regional hospital	0 (0%)	0 (0%)	3 (60%)	4 (9.8%)	0 (0%)	14%
Tertiary hospital	1 (100%)	1 (100%)	2 (40%)	18 (44%)	4 (100%)	77%
If an NCD patient arrives at the referred facility, are you normally informed?						
Yes	0 (0%)	0 (0%)	0 (0%)	3 (7.3%)	1 (25%)	6%
No	1 (100%)	1 (100%)	5 (100%)	38 (93%)	3 (75%)	96%
Are patient records transferred back to this facility?						
Yes	0 (0%)	0 (0%)	0 (0%)	5 (12%)	1 (25%)	7%
No	1 (100%)	1 (100%)	5 (100%)	36 (88%)	3 (75%)	93%
Does this facility receive NCD-related referrals from screening and/or surveillance activities conducted in the community?						
Yes	1 (100%)	1 (100%)	2 (40%)	20 (49%)	2 (50%)	68%
No	0 (0%)	0 (0%)	3 (60%)	21 (51%)	2 (50%)	32%
Overall mean readiness	67%	56%	40%	47%	56%	53%

**Domain 7:** Facility infrastructure. Overall mean readiness for each facility level was calculated by averaging the percentage of facilities responding ‘Yes’ across all items within this domain (n = 19 items).

Item	Referral hospital, *N* = 1	Regional hospital, *N* = 1	District hospital, *N* = 5	Primary health care center, *N* = 41	Parastatal, *N* = 4	Mean readiness
Availability of water supply during the past 7 days						
Always available	100%	100%	100%	85%	100%	97%
Interruptions of more than 2 h per day	0%	0%	0%	15%	0%	3%
Availability of functioning toilet						
Yes	100%	100%	100%	98%	100%	100%
No	0%	0%	0%	2%	0%	0%
Availability of onsite pharmacy						
Yes	100%	100%	100%	88%	100%	98%
No	0%	0%	0%	12%	0%	2%
Reliable method of drug storage						
Yes	100%	100%	100%	93%	100%	99%
No	0%	0%	0%	7%	0%	1%
Availability of power supply						
National or community grid	100%	100%	100%	100%	100%	100%
Generator	100%	100%	100%	2%	50%	70%
Solar system	100%	0%	20%	17%	25%	32%
Invertor	0%	0%	0%	0%	0%	0%
Battery	100%	0%	0%	0%	0%	20%
Functional ambulance or other vehicle for emergency transportation						
Yes	100%	100%	100%	5%	100%	81%
No	0%	0%	0%	95%	0%	19%
Availability of fuel today						
Yes	100%	100%	80%	0%	100%	76%
No	0%	0%	20%	98%	0%	24%
Don’t know	0%	0%	0%	2%	0%	0%
Regularity of fuel availability						
Routinely	100%	NA%	0%	100%	100%	75%
Periodically	0%	NA%	100%	0%	0%	25%
Functioning landline telephone						
Yes	100%	0%	0%	2%	50%	30%
No	0%	100%	100%	98%	50%	70%
Functioning cellphone						
Yes	0%	0%	0%	20%	75%	19%
No	100%	100%	100%	80%	25%	81%
Functioning short wave radio						
Yes	0%	0%	0%	0%	50%	10%
No	100%	100%	100%	100%	50%	90%
Functioning laptop/computer						
Yes	100%	100%	100%	88%	100%	98%
No	0%	0%	0%	12%	0%	2%
Internet services						
Yes	100%	100%	100%	10%	100%	82%
No	0%	0%	0%	90%	0%	18%
Accessibility of internet over the past 7 days						
Yes	100%	100%	60%	10%	100%	74%
No	0%	0%	40%	90%	0%	26%
Overall mean readiness	89%	65%	64%	45%	81%	69%

**Domain 8:** Financing and payment mechanism. Overall mean readiness for each facility level was calculated by averaging the percentage of facilities responding ‘Yes’ across all items within this domain (n = 11 items, covering health insurance participation, charging practices, receipt of funds for inputs, and facilities reporting no external funds).

	Referral hospital, *N* = 1	Regional hospital, *N* = 1	District hospital, *N* = 5	Primary health care center, *N* = 41	Parastatal, *N* = 4	Mean readiness
Does this facility participate in a health insurance scheme?						
Yes	1 (100%)	0 (0%)	0 (0%)	0 (0%)	3 (75%)	35%
No	0 (0%)	1 (100%)	5 (100%)	41 (100%)	1 (25%)	65%
Number of facilities charging for selected services						
Consultation	0 (0%)	0 (0%)	0 (0%)	0 (0%)	3 (75%)	15%
Laboratory tests	0 (0%)	0 (0%)	0 (0%)	0 (0%)	4 (100%)	20%
X-rays and other imaging	0 (0%)	0 (0%)	0 (0%)	0 (0%)	3 (75%)	15%
Supplies (e.g., compresses, syringes, etc.)	0 (0%)	0 (0%)	0 (0%)	0 (0%)	2 (50%)	10%
Medicines	0 (0%)	0 (0%)	0 (0%)	0 (0%)	4 (100%)	20%
Number of facilities receiving funds for input						
Administration	0 (0%)	1 (100%)	2 (40%)	0 (0%)	2 (50%)	38%
Supplies	1 (100%)	0 (0%)	2 (40%)	0 (0%)	1 (25%)	33%
Equipment	1 (100%)	0 (0%)	2 (40%)	1 (2.4%)	2 (50%)	38%
Medicine	1 (100%)	0 (0%)	2 (40%)	0 (0%)	2 (50%)	38%
None	0 (0%)	0 (0%)	3 (60%)	40 (98%)	1 (25%)	37%
Overall mean readiness	67%	8%	13%	0%	66%	31%

## Annex 2: mapping of readiness domains and tracer items to source frameworks

**Table:**

Readiness domain	Example tracer items	Primary source framework	Notes
Human resources	GP, nurses, pharmacists, cardiologists, cardiac surgeons	WHO SARA + PEN-Plus adaptation	Some specialist cadres may exceed routine PHC staffing expectations
Essential medicines	Metformin, insulin, ACE inhibitors, inhalers	WHO SARA/WHO PEN	Standard NCD medicine indicators
Diagnostics & equipment	BP machine, glucometer, ECG, ultrasound cardiac probe	WHO SARA + PEN-Plus	Some advanced diagnostics not routinely expected at PHC level
Infrastructure	Electricity, water, internet, ambulance access	WHO SARA	Standard facility readiness indicators
Referral & follow-up systems	Referral feedback, patient tracing, follow-up systems	ABCE + locally adapted items	Added to assess continuity of chronic care
Financing mechanisms	Insurance participation, user fees, out-of-pocket costs	ABCE	Added to assess financial protection dimensions
Health education & support	Educational materials, community programs, social support	WHO PEN/locally adapted	Focused on patient-centered NCD care
